# Regulation of early growth response 2 expression by secreted frizzled related protein 1

**DOI:** 10.1186/s12885-017-3426-y

**Published:** 2017-07-07

**Authors:** Kelly J. Gregory, Stephanie M. Morin, Sallie S. Schneider

**Affiliations:** 1 Pioneer Valley Life Sciences Institute, Baystate Medical Center, 3601 Main St, Springfield, MA 01199 USA; 2 Department of Biology, University of Massachusetts, Amherst, MA 01003 USA; 3 Veterinary and Animal Sciences, University of Massachusetts, Amherst, MA 01003 USA

**Keywords:** SFRP1, EGR2, TGF-β, MAPK, Mammary gland, Macrophage polarization

## Abstract

**Background:**

Secreted frizzled-related protein 1 (SFRP1) expression is down-regulated in a multitude of cancers, including breast cancer. Loss of *Sfrp1* also exacerbates weight gain as well as inflammation. Additionally, loss of SFRP1 enhances TGF-β signaling and the downstream MAPK pathway. TGF-β has been shown to increase the expression of Early Growth Response 2 (EGR2), a transcription factor implicated in immune function in a wide variety of cell types. The work described here was initiated to determine whether SFRP1 modulation affects TGF-β mediated EGR2 expression in mammary tissues as well as macrophage polarization.

**Methods:**

Real-time PCR analysis was performed to examine *EGR2* expression in human and murine mammary epithelial cells and tissues in response to SFRP1 modulation. Chemical inhibition was employed to investigate the roles TGF-β and MAPK signaling play in the control of EGR2 expression in response to SFRP1 loss. Primary murine macrophages were isolated from S*frp*1^−/−^ mice and stimulated to become either M1 or M2 macrophages, treated with recombinant SFRP1, and real-time PCR was used to measure the expression of murine specific M1/M2 markers [Egr2 (M2) and Gpr18 (M1)]. Immunohistochemical analysis was used to measure the expression of human specific M1/M2 markers [CD163 (M2) and HLA-DRA (M2)] in response to rSFRP1 treatment in human mammary explant tissue.

**Results:**

Knockdown of SFRP1 expression increases the expression of *EGR2* mRNA in human mammary epithelial cells and addition of rSFRP1 decreases the expression of *EGR2* when added to explant mammary gland tissues. Chemical inhibition of both TGF-β and MAPK signaling in *Sfrp*1^−/−^ or knockdown mammary epithelial cells results in decreased expression of *EGR*2. Stimulated murine macrophages obtained from S*frp*1^−/−^ mice and treated with rSFRP1 exhibit a reduction in *Egr2* expression and an increase in *Gpr18* mRNA expression. Human mammary explant tissue treated with rSFRP1 decreases CD163 protein expression whereas there was no effect on the expression of HLA-DRA.

**Conclusions:**

Loss of SFRP1 likely contributes to tumor progression by altering the expression of a critical transcription factor in both the epithelium and the immune system.

**Electronic supplementary material:**

The online version of this article (doi:10.1186/s12885-017-3426-y) contains supplementary material, which is available to authorized users.

## Background

The Secreted Frizzled Related Proteins (SFRPs) encode a family of secreted proteins with a cysteine-rich domain homologous to the Wnt-binding domain of FZD receptor proteins [1]. Expression of SFRP family members antagonize Wnt signaling by binding to Wnt ligands and preventing ligand-receptor interactions and signal transduction [2]. SFRP1 is a member of this protein family and is significantly down-regulated in breast tumors and in breast carcinoma cell lines [3, 4]. When SFRP1 is re-expressed in tumor cells, invasion and cellular proliferation is suppressed suggesting that it is an important tumor suppressor protein. More recent data has suggested that SFRP1 may also play an important role in controlling inflammation [5–7]. Wnt5a is expressed by activated antigen presenting cells in rheumatoid arthritis joints and stimulates the expression of cytokine expression including Interleukin (IL)-1, IL-6 and IL-8 through the Fzd5- CamKII non-canonical Wnt signaling pathway [8, 9]. SFRP1 can block this process [6, 10] and can inhibit leukocyte activation and cytokine production in vitro [5] as well as reduce neutrophil infiltration in ischemic tissue in vivo [11]. Conversely, a targeted deletion of *Sfrp1* has been demonstrated to increase obesity-induced macrophage infiltration in murine mammary glands and fat depots [12].

Early Growth Response 2 (EGR2) is a zinc-finger transcription factor of the early growth response gene (EGR) family [13], which regulates gene expression by binding to *cis*-acting elements of the target genes [14, 15]. EGR2 is known to carry out essential functions in hindbrain development as well as myelination of the peripheral nervous system [16, 17] and also plays an important role in regulating inflammatory autoimmunity and antigen receptor-mediated lymphocyte proliferation [18]. Interestingly, Egr2 was recently identified as a novel marker which identifies a specialized subset of murine macrophages termed M2 polarized macrophages [19].

Considering the association between SFRP1 loss and increased macrophage involvement in rodents, we sought to determine whether SFRP1 modulation in human mammary tissues affects EGR2 expression and macrophage polarization. Our data reveal that the expression of SFRP1 is inversely related to the expression of EGR2 in human mammary and mouse epithelial cells and tissues. Additionally, we provide evidence that SFRP1 loss modulates EGR2 partially through a TGF-β and MAPK dependent pathway. Finally, we clearly demonstrate that rSFRP1 treatment affects macrophage polarization in *Sfrp*1^−/−^ derived macrophages and human mammary gland explant cultures. Taken together, these results highlight a novel pathway by which extracellular SFRP1 can control the pro-tumorigenic niche.

## Methods

### Animals

This study was carried out in strict accordance with the recommendations in the Guide for the Care and Use of Laboratory Animals of the National Institutes of Health. The protocol was approved by the Baystate Medical Center Institutional Animal Care and Use Committee (Permit Number: 132-681). Four week old female BALB/c control mice (*n* = 30) and BALB/c *Sfrp1* ^−/−^ mice (*n* = 22) were individually housed in plastic cages with food and water provided continuously, and maintained on a 12:12 light cycle. The animals were treated with DMBA by gavage (1 mg/week) for 4 consecutive weeks to induce mammary tumor formation. Tumor size, number, and time-to-incidence was noted and compared between control and *Sfrp1* ^−/−^ animals. Tumors were collected from 9 control mice and 7 *Sfrp1* ^−/−^ mice, flash frozen and stored at −80 °C until processed for RNA isolation.

### Human cell and explant culture

The 76 N TERT cell line (obtained from Dr. Vimla Band) were stably transfected with either pSUPER.retro (TERT-pSUPER) or siSFRP1-PSUPER.retro (TERT-siSFRP1) and cultivated as previously described [20]. MCF7, T47D, and MDA-MB-231 cells were purchased from ATCC (ATTC#s HTB-22, HTB-133, and HTB-26) and TMX2–28 cells [21] were obtained from Dr. Kathleen Arcaro. Breast cancer cell lines were transfected with either pCDNA3.1 or SFRP1-pCDNA3.1 as previously described [22]. Cells were grown to 70% confluence in 6-well plates for RNA isolation. For some experiments, cells were treated with DMSO, 10 μM LY364947 (L6293; Sigma), 5 μM U0126 (U120; Sigma), or 10 μM FR108204 (sc203945; Santa Cruz Biotechnology) 24 h prior to RNA isolation. Fresh breast tissue enriched with epithelium from women undergoing elective breast surgery was grossly dissected from the surrounding adipose tissue and placed on Surgifoam gelatin sponges (Ferrosan, Sueborg, Denmark) in 60 mm tissue culture dishes containing 3 mL of medium [(phenol red free DMEM/F12 buffered with Hepes and NaHCO_3_ from Gibco (Invitrogen, Carlsbad, CA)], 5 μg/mL human insulin, 1X antibiotic/antimycotic (100 U/mL penicillin/streptomycin and 0.250 μg/mL amphotericin B), and 10 μg/mL gentamycin from Sigma (Sigma, St. Louis, MO). The media was supplemented with either 0.1% BSA or 1 μg/ml rSFRP1 for 24 h and the tissue was subsequently flash frozen and stored at −80 °C until being processed for RNA isolation.

### Primary mouse mammary epithelial and macrophage cell culture

Ten week old virgin control (*n* = 12) or *Sfrp1* ^−/−^ mice (*n* = 12) were euthanized with carbon dioxide prior to organ removal. The fourth mammary glands were harvested, minced, and finally dissociated in DMEM:F12 (Sigma) supplemented with 5% fetal bovine serum (Gibco, Waltham, MA), 2 mg/ml collagenase (Worthington Biochemical, Freehold, NJ), 100u/ml hyaluronidase (Sigma), 100u/ml pen/strep (Gibco) and 100 μg/ml gentamicin (Gibco) for 6 h. The cell pellet was collected and further dissociated with 1 ml pre-warmed 0.05% Trypsin-EDTA (Gibco) and l0 1 mg/ml DNase I (Roche, Mannheim, Germany). Cell suspensions were sequentially sieved through 100 μm and 40 μm cell strainers. Primary cells were seeded onto rat tail collagen-1 (BD Biosciences, San Jose, CA) coated tissue culture dishes in 10% serum containing mammary growth medium (EpiCult®B for Mouse Mammary Epithelial Cell Culture, Vancouver, BC) supplemented with10ng/ml EGF (Sigma), 10 ng/ml FGF (Sigma), 4 μg/ml heparin, 100u/ml pen/strep (Gibco) and 100 μg/ml gentamicin (Gibco) [23]. Cells were routinely cultivated at 37 °C in 5% CO_2_. Serum containing media was removed the next day and replaced with media containing DMSO, 10 μM LY364947, 5 μM U0126, or 10 μM FR108204 24 h prior to RNA isolation. The spleens from *Sfrp1* ^−/−^ mice were removed aseptically, placed in 100-mm^2^ tissue culture dishes with 5 ml of phosphate buffered saline (PBS) and the cellular contents was released by macerating the spleens between frosted glass slides. The cells were collected by centrifugation, re-suspended in RPMI media (Gibco), plated in 6-well plates, and stimulated with either LPS (Sigma) or 2.5 ng/mL TGFβ1 (Sigma) and subsequently treated with either 0.1% BSA or 1 μg/ml rSFRP1. The following day, the media was removed and the adherent macrophage rich cells were harvested for RNA isolation.

### RNA isolation and real-time PCR analysis

Total RNA was extracted from cells and tissues (*n* = 3/treatment) using an acid-phenol extraction procedure [24], according to the manufacturer’s instructions (Trizol, Invitrogen, Carlsbad, CA). Relative expression levels of mRNA was determined by quantitative real-time PCR using the Mx3005P® real-time PCR system (Agilent, Santa Clara, CA) and all values were normalized to the amplification of *ΑctB.* The PCR primer sequences for mouse *Actb*, mouse *Tgfb*1, and human *ACTB* have been published [12, 20]. Additionally, primer sequences were designed to cross exon junctions using GenScript Real-time PCR Primer Design (www.genscript.com/tools/real-time-pcr-tagman-primer-design-tool) and are as follows: human *EGR2* forward: 5′-TCCCAGTAACTCTCAGTGGTT-3′, human *EGR2* reverse: 5-TGCCATCTCCGGCCA-3′; mouse *Egr2* forward: 5′- TTGACCAGATGAACGGAGTG–3′, mouse *Egr2* reverse: 5′-AGCTACTCGGATACGGGAGA–3′; mouse *Gpr*18 forward: 5′- TGTCAACGTGCTCAACTTCA–3′, mouse *Gpr*18 reverse: 5′- CCTTGGGCTTCAGCTTAGA–3′; human *CD*163 forward: 5′-GAGTGACCTGCTCAGATGGA–3′, human *CD*163 reverse: 5′-CCGTCCTTGGAATTTGATCT–3′; human *HLADRA* forward: 5′- CATGGAGGTGATGGTGTTTC–3′, human *HLADRA* reverse: 5′- TGCTTTCACTGAGGTCAAGG–3′. The assays were performed using the 1-Step Brilliant® SYBRIII® Green QRT-PCR Master Mix Kit (Agilent) containing 200 nM forward primer, 200 nM reverse primer, and 100 ng total RNA. The conditions for cDNA synthesis and target mRNA amplification were performed as follows: 1 cycle of 50 °C for 30 min;1 cycle of 95 °C for 10 min; and 35 cycles each of 951C for 30 s, 55 °C for 1 min, and 72 °C for 30 s. Non-template controls were included to control for primer dimers and no reverse-transcriptase controls were included to control for genomic DNA amplification.

### Western blot analysis

Treated MMECs were washed twice with cold PBS and 100 μL of cold lysis buffer [50 mM Tris-HCl, 150 mM NaCl, 100 mM NaF, 10 mM MgCl_2_, 0.5% NP40, protease inhibitor cocktail, and phosphatase inhibitor I and II (Sigma)] was added directly to the plate. The cells were incubated for 30 min at 4 °C on a shaker and then harvested using a rubber policeman. The lysates were passed 4 times through a 26 gauge syringe, kept on ice for 30 min, and then centrifuged for 20 min at 12,000 rpms at 4 °C. The supernatant was transferred to a new tube and the protein was quantified utilizing the BCA™ Protein Assay Kit (Pierce, Rockford, IL). A total of 30 μg of protein was run on a 10% SDS-Page gel and transferred to a PVDF membrane. The membrane was blocked for 45 min with 5% milk in tris-buffered saline containing 0.05% Tween-20 (TBS-T). The primary antibodies used in this study were [Rabbit phospho-ERK1/2 (Thr202/Tyr204) (1:1000), #4377, Cell Signaling Technologies, Danvers, MA and Rabbit β-actin (1:1000), ab8227, Abcam, Cambridge, MA] incubated overnight at 4 °C. The secondary antibody [anti-rabbit IgG-HRP (#7074, Cell Signaling Technologies) was applied (1:1000) and incubated for 45 min at room temperature. The blot was washed and developed using a Western Blot Luminol Reagent (Denville Scientific, Holliston, MA). The integrated band densities were measured using ImageJ software (www.imagej.nih.gov).

### Immunohistochemistry

Tissue blocks were sectioned at 4 μm on a graded slide, deparaffinized in xylene, rehydrated in graded ethanols, and rinsed in phosphate-buffered saline (PBS). Immunohistochemistry (IHC) was performed on a DakoCytomation autostainer using the Envision HRP Detection system (Dako, Carpinteria, CA). Each mammary tissue block was sectioned at 4 μm on a graded slide, deparaffinized in xylene, rehydrated in graded ethanols, and rinsed in Tris-phosphate-buffered saline (TBS). Heat induced antigen retrieval was performed in a microwave at 98 °C in 0.01 M citrate buffer. After cooling for 20 min, sections were rinsed in TBS and subjected to the primary mouse monoclonal anti-CD163 [GH1/61] antibody (1:100, Abcam, ab111250) or the primary rabbit polyclonal anti-HLA-DR antibody (1:250, Abcam, ab137832) for 30 min. Immunoreactivity was visualized by incubation with chromogen diaminobenzidine (DAB) for 5 min. Tissue sections were counterstained with hematoxylin, dehydrated through graded ethanols and xylene, and cover-slipped. Images were captured with an Olympus BX41 light microscope using SPOT Software 5.1 (SPOT™ Imaging Solutions, Detroit, MI).

### Statistical analysis

Group means were compared using Student’s t-tests (Graphpad Prism) and results with *P* < 0.05 were considered significant. A test for outliers was performed on all data sets using a Grubbs’ test (GraphPad QuickCalcs) and statistical outliers were not included data analysis.

## Results

### SFRP1 alters the transcriptional regulator EGR2 in human and murine mammary epithelial cells and tissues.

**Fig. 1:**
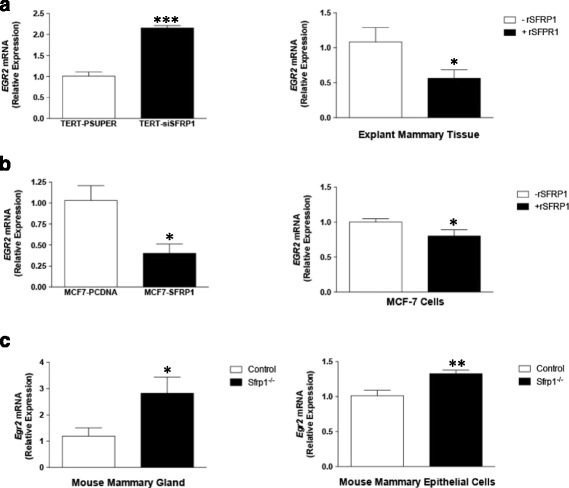
SFRP1 alters the expression of EGR2 in human and mouse mammary epithelial cells and tissues. **a** Total RNA was isolated TERT-pSUPER and TERT-siSFRP1 cell lines (*left panel*) and from human explant cultures treated in the absence and presence of rSFRP1 (*right panel*) for real-time PCR analysis of *EGR*2 mRNA expression. **b** Total RNA was isolated MCF7-PCDNA and MCF7-SFRP1 cell lines (*left panel*) and MCF7 cells treated in the absence and presence of rSFRP1 (*right panel*) for real-time PCR analysis of EGR2 mRNA expression. **c** Total RNA was isolated from the mammary glands of 10 wk. virgin control and *Sfrp1* ^−/−^ female mice (*left panel*) and mouse mammary epithelial cells derived from control and *Sfrp1* ^−/−^ mice (*right panel*) for real-time PCR analysis of *Egr*2 expression. Bars represent mean ± SEM *EGR2/ACTB* and are expressed as relative expression of control groups. *p < 0.05, **p < 0.01, ***p < 0.01 (significantly different from control using student’s *t*-test)

*EGR2* is expressed in breast cancer cell lines, particularly the more aggressive triple negative subtypes and may be regulated in part by Epidermal Growth Factor (EGF) family members [18, 25]. Considering that EGR2 and the tumor suppressor protein SFRP1 and play a role in immune function, we sought to determine whether SFRP1 regulates *EGR2* expression. We found that TERT-siSFRP1 cells express significantly more *EGR*2 mRNA when compared with TERT-pSUPER cells and when human explant mammary tissues are treated with rSFRP1, *EGR*2 mRNA levels are significantly reduced (Fig. 1a). Conversely, breast cancer cells overexpressing SFRP1 (MCF7-SFRP1) express less *EGR*2 when compared with vector transfected cells (MCF7-pCDNA) and exogenous rSFRP1 treatment reduces *EGR2* expression in MCF7 cells (Fig. 1b). When we further tested the effect of SFRP1 expression in a panel of breast cancer cell lines, we found that SFRP1 reduced the mRNA levels of *EGR2* in T47D cells but not in TMX2–28 cells (a Tamoxifen resistant variant of MCF7) or MDA-MB-231 cells (Additional file 1: Figure S1).

We next sought to establish whether mouse mammary gland tissue derived from S*frp*1^−/−^ mice also exhibit an increase in *Egr2* expression. We found that when compared with control mice, mammary tissue from S*frp*1^−/−^ mice have elevated levels of *Egr*2 (Fig. 1c, *left panel)* and more specifically, isolated mouse mammary epithelial cells (MMECs) from S*frp*1^−/−^ mice express higher levels of *Egr*2 (Fig. 1c, *right panel).*

### Regulation of EGR2 by TGF-β and MAPK signaling in human and murine mammary epithelial cells

**Fig. 2:**
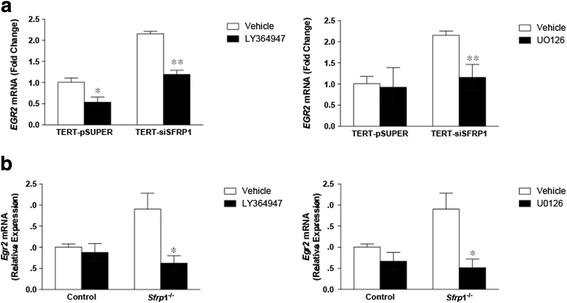
The expression of *EGR*2 is regulated by TGF-β and MAPK signaling in human and mouse mammary epithelial cells with reduced SFRP1 expression. **a** TERT-pSUPER and TERT-siSFRP1 cells were treated with 10 μM LY364947 (*left panel*), or 5 μM U0126 (*right panel*), for 24 h and total RNA was isolated from three separate harvests for real-time PCR analysis of *EGR*2. **b** Mouse mammary epithelial cells were treated with 10 μM LY364947 (*left panel*), or 5 μM U0126 (*right panel*), for 24 h and total RNA was isolated from three separate harvests for real-time PCR analysis of *Egr*2. All real-time PCR results are from two separate experiments performed in triplicate and results were normalized to amplification of *ACTB* mRNA. Bars represent mean ± SEM and are expressed as fold change with respect to TERT-pSUPER cells or control MMECs. *p < 0.05, **p < 0.01 (significantly different from DMSO treated group using student’s *t*-test)

The expression of EGR2 is regulated by TGF-β signaling in skin fibroblasts [26] and by MAPK signaling in osteoblasts and breast adipose fibroblasts [27–29]. As we have previously demonstrated that reducing SFRP1 in immortal mammary epithelial cells exacerbates TGF-β signaling and increases migration through TGF-β mediated MAPK signaling [30], we suspected that SFRP1 mediated modulation of *EGR2* may involve these pathways. In TERT-siSFRP1 cells, we confirmed that *EGR*2 expression is upregulated in response to TGF-β1 treatment (Additional file 2: Figure S2A). We next sought to determine whether *EGR2* expression in TERT-siSFRP1 cells could be blocked by antagonizing the TGF-βR with LY364947. We found that both TERT-pSUPER and TERT-siSFRP1 cells exhibited a significant reduction in *EGR*2 mRNA expression when the TGF-βR is inhibited (Fig. 2a, *left panel*)*.* Considering that TGF-β signaling induces ERK1/2 activation [31] and loss of SFRP1 exacerbates the MAPK pathway [30], we next tested whether *EGR2* expression could be affected in response to a MEK1/2 specific inhibitor (U0126). We clearly demonstrate that while the expression of *EGR*2 is not affected by U0126 in TERT-pSUPER cells, MEK1/2 inhibition in TERT-siSFRP1 cells significantly decreased *EGR*2 expression (Fig. 2a, *right panel*). Moreover, TERT-siSFRP1 cells treated with a second more specific MEK1/2 inhibitor, FR108204, exhibit a decrease in *EGR2* expression when compared with the effect of FR108204 on EGR2 mRNA in TERT-pSUPER cells (Additional file 3: Figure S3A).

**Fig. 3:**
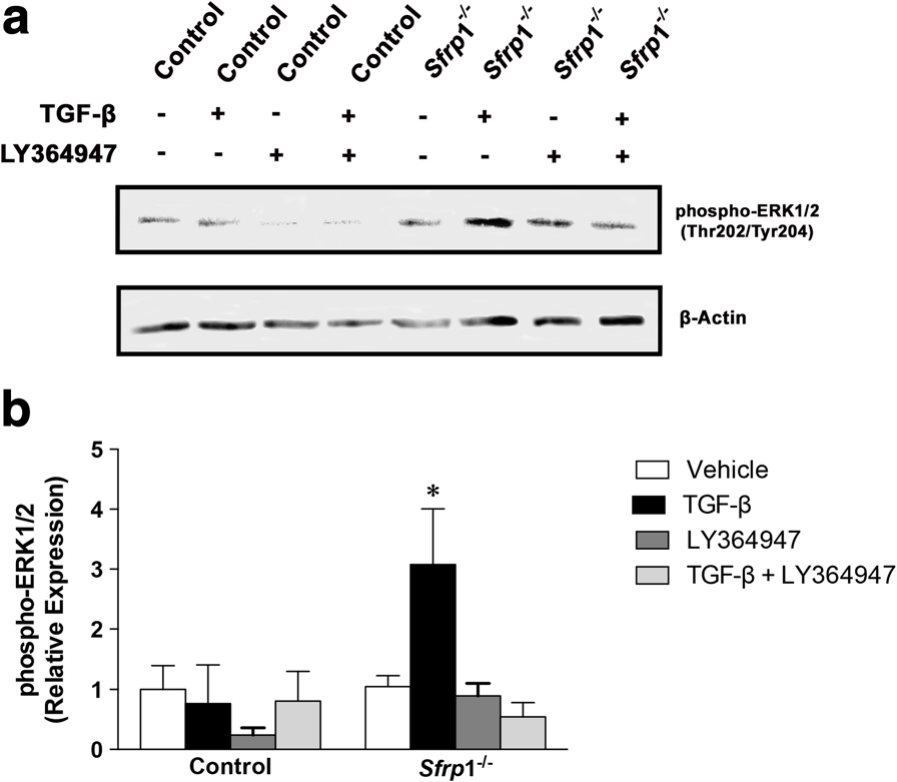
Loss of SFRP1 increases TGF-β mediated ERK1/2 activation in MMECs. **a** Control and *Sfrp1* ^−/−^ MMECs were treated with 2.5 ng/ml TGF-β1 and/or 10 μM LY364947 in triplicate wells and cell lysates were analyzed for phospho-ERK1/2 and β-Actin protein expression by western blot. **a** Image of representative western blot illustrating band densities in response to treatment and **b** quantification of integrated band densities from three separate western blots. *p < 0.05, (significantly different from vehicle treated cells using a student’s *t*-test)

An *Sfrp*1^−/−^ associated increase in TGF-β expression has previously been confirmed in our analysis of pubertal rodent mammary tissues [12, 32]. We next wanted to determine whether similar to human cells, treatment with TGF-β1 could induce the phosphorylation of ERK1/2 in MMECs and if the phosphorylation of ERK1/2 could be blocked by antagonizing the TGF-βR with LY364947. Our results illustrate that TGF-β1 treatment in control MMECs does not affect ERK1/2 phosphorylation, but blocking the TGF-βR abrogates phosphorylation. However, MMECs derived from *Sfrp*1^−/−^ mice exhibited a significant increase in ERK1/2 phosphorylation in response to TGF-β1 treatment, which was also blocked by LY364947 treatment (Fig. 3). To establish whether there is a connection between TGF-β1 signaling and *Egr2* in our murine model, we used MMECs from our control mice to verify that *Egr*2 expression in MMECs is driven by TGF-β treatment (Additional file 2: Figure S2B). Consistent with our human mammary epithelial SFRP1 knockdown cells, we show that TGF-βR, MEK1/2, and ERK1/2 inhibition reduce *Egr2* expression in *Sfrp*1^−/−^ MMECs (Fig. 2b; Additional file 3: Figure S3B).

### The effect of rSFRP1 treatment on M1 and M2 polarization in Sfrp1^−/−^ derived macrophages and human mammary gland explant cultures.

**Fig. 4:**
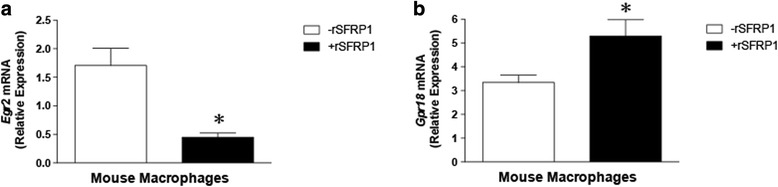
The effect of rSFRP1 treatment on M1 and M2 polarization in *Sfrp1* ^−/−^ dervived macrophages. Macrophages were isolated from spleens derived from *Sfrp1* ^−/−^ mice, treated in the presence and absence of rSRP1, and stimulated with either TGF- β1 or LPS (**b**) for 24 h. M2 polarization was evaluated by the mRNA expression of *Egr*2 and M1 polarization was evaluated by *Grp*18 mRNA expression. The results shown represent experiments performed in duplicate and normalized to the amplification of *Actb* mRNA. Bars represent mean ± SEM of the fold change with respect to control mice. *p < 0.05, **p < 0.01 (significantly different from vehicle treated macrophages using student’s *t*-test)

We previously reported that a targeted deletion of *Sfrp1* exacerbates weight gain as well as inflammation [12]. The increased macrophage infiltration and pro-inflammatory cytokine expression observed in S*frp*1^−/−^ mice was expected based on the link between obesity and inflammation. The assortment of cues within the microenvironment can elicit a wide range of macrophage phenotypes and functions [33]. The classical (M1) and alternative (M2) activation of macrophage subtypes are an example of the two extremes on this continuum. Interestingly, Gong et al. have demonstrated that TGF-β signaling is required for M2 activation [7] and Egr2 is also murine marker of M2-polarized macrophages [19]. Therefore, we next tested the hypothesis that SFRP1 may play a role in macrophage polarization. We isolated splenic macrophages from S*frp*1^−/−^ mice and treated them with either TGF-β to induce M2 polarization or LPS to induce M1 polarization. When TGF-β stimulated macrophages were treated with rSFRP1, the M2 maker *Egr*2 was significantly down regulated (Fig. 4a). Conversely, when LPS stimulated macrophages were treated with rSFRP1, the M1 marker *Grp*18 was significantly up-regulated (Fig. 4b).

**Fig. 5:**
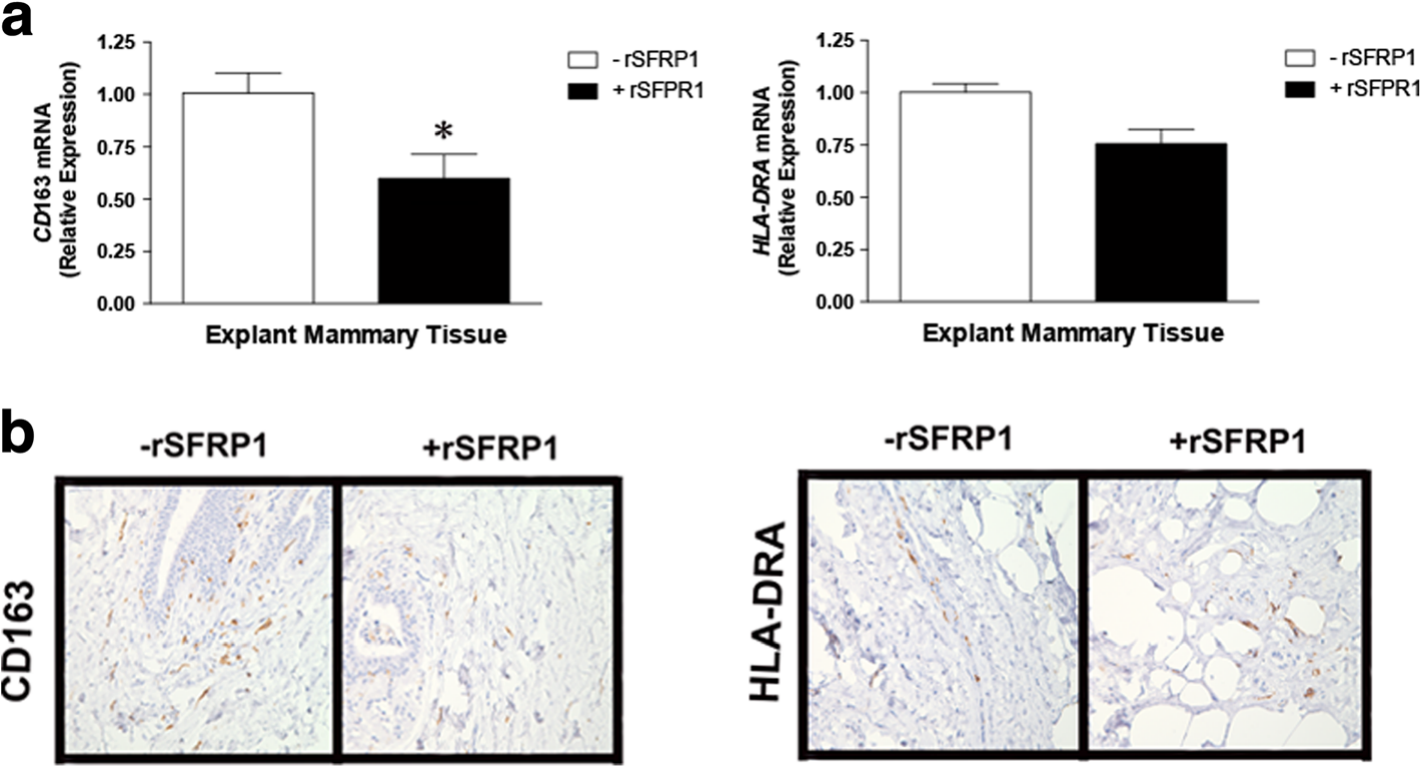
The effect of rSFRP1 treatment on M1 and M2 polarization in human mammary gland explant cultures. **a** Total RNA was isolated from explant cultures dervived from normal human breast tissue treated in the absence and presence of 1 μg/ml rSFRP1. **a** M2 polarization was evaluated by the mRNA expression of *CD*163 (*left pana*l) and M1 polarization was evaluated by *HLA-DRA* mRNA expression (*right panal*). **b** Explant mammary gland sections were subjected to immunohistochemical analysis, stained for CD163 (*left pane*l) or HLA-DRA (*right panel)* and images were captured at 100X. Representative pictures are displayed for tissues from each treatment group which was performed in triplicate samples. *p < 0.05 (significantly different from untreated mammary tissue using student’s *t*-test)

Considering that rSFRP1 has been used to decrease IL-6 in macrophages and adipocytes [12, 34] and TGF-β stimulated macrophages treated with rSFRP1 exhibit a reduction in *EGR2* expression, we investigated whether rSFRP1 treatment of a human mammary explant would affect the expression of the human M2 marker, CD163. Human breast explant cultures were treated with rSFRP1 for 24 h and were subsequently harvested for real-time PCR analysis as well as immunohistochemistry. We show that in response to rSFRP1, the mRNA levels and number of CD163 staining cells are significantly downregulated in the human breast tissue (Fig. 5a). However, HLA-DR expression (a human M1 marker) was not altered by rSFRP in human mammary gland explant cultures (Fig. 5b).

### Discussion

Previous studies have noted that SFRP1 can elicit anti-inflammatory effects [5, 6, 35]. The work described here confirms the regulatory role of SFRP1 on TGF-β and its effect on EGR2 not only in inflammatory cells, but also in epithelial cells. Our data suggests that the control of inflammation may be due in part to the concomitant effect of SFRP1 on *EGR* expression. Several EGR family members have been implicated in regulation of cytokine expression in allergic reactions, mesenchymal stem cells as well as prostate cancer cells [18, 36, 37]. Moreover, EGR2 plays a complex role in a variety of cell types as well as the development of cancer. In Ras transformed NIH 3 T3 cells it controls *cebpb* expression [38] in gastric cancer cells its expression is associated with metastasis [39] and inversely associated with the expression of miR20a [40]. Knockdown of EGR2 in leiomyoma cells increased *myc* and *PCNA* expression as well as collagen deposition [41]. However, how EGR2 contributes to breast cancer however is not clear, its expression has been suggested to drive both the expression of both *Erbb2*, as well as aromatase expression [25, 29, 42]. The data presented here expand upon these findings and demonstrate that SFRP1 regulates EGR2 expression in both human and murine mammary epithelium. Interestingly, SFRP1 does not affect EGR2 expression in estrogen receptor (ER) negative cells (TMX2–28 and MDA-MB-231) which hints at a role ER signaling may play in EGR2 regulation. We have previously shown that ER signaling is upregulated in response to SFRP1 loss in both human and mouse tissues [22] which further supports the hypothesis that ER signaling may be involved in EGR2 expression. Data presented by Windahl et al. demonstrate that when one of the activation functions (AF1) within the ER gene is disrupted in mice, osteoblasts exhibit a blunted *Egr2* mRNA expression in response to mechanical strain [43]. Taken together, more research is required in order to elucidate the role ER plays in EGR2 regulation.

TGF-β and Wnt signaling regulate a variety of physiological processes including mammary gland development and tumorigenesis. SFRP1 has been associated with the control of TGF-β and Wnt signaling. Specifically, *Sfrp*1^−/−^ derived mammary tissues express increased levels of *Tgfb*1 and Wnt4 mRNA and TERT-siSFRP1 cells are more sensitive to TGF-β and Wnt signaling [30, 32]. Fang et al. was the first group to reveal that Egr2 is a transcriptional target of TGF-β [26]. Here we demonstrate that TGF-β stimulates and TGFBR inhibition represses *EGR2* mRNA expression in both HMECs as well as MMECs (Additional file 2: Figure S2, Fig. 2). We have also observed that tumors derived from *Sfrp*1^−/−^ mice express significantly higher levels of both *Tg*f*b*1 and *Egr*2 mRNA (data not shown). Dillon et al. revealed that Egr2 expression is upregulated in Erbb2 driven mammary tumors [25]. However, these researchers did not evaluate the role TGF-β plays in their model of tumorigenesis and therefore future research will be directed identifying how loss of SFRP1 together with TGF-β1, EGR2 upregulation, and additional tumor initiating pathways promote mammary carcinogenesis.

We have previously shown that activated ERK1/2 levels and the migratory action of TERT-siSFRP1 cells are drastically reduced in response to TGF-βR inhibition which is consistent with work described by Imamichi et al. showing that TGF-β signaling mediates the cellular migration of breast cancer cells by ERK1/2 activation [31]. Our current data shows that inhibition MAPK signaling in *Sfrp*1^−/−^ murine mammary glands or knockdown mammary epithelial cells results in decreased expression of *EGR*2. These findings are consistent with findings described by Zaman et al. showing that MEK1/2 inhibition the osteoblast UMR106 cell line resulted in decreased EGR2 expression (26). Chandra et al. demonstrate that the MAPK/ERK pathway is a major downstream signaling pathway mediating the stimulatory effects of EGF on EGR2 expression and osteoprogenitor survival [28]. Finally, To et al. report that the same MEK inhibitor utilized in our experiments, U0126, was the elicited the most potent inhibition of *EGR*2 transcription in breast adipose fibroblasts [29].

Macrophage polarization is an occurrence that spans two extremes from the classically activated M1 macrophages to the alternatively activated M2 macrophages. M1-type macrophages inhibit cancer development, while M2-type macrophages stimulate wound healing and are associated with cancer growth and proliferation. Our data reveal that mouse macrophages polarized to M1 macrophages in response to LPS exhibit a significant increase in M1 marker expression when treated with recombinant SFRP1. The fact that we did not observe similar findings when human mammary gland explants were treated with SFRP1 could be due to the fact that our murine macrophages were isolated from mice with no endogenous *Sfrp1* and the expression levels of SFRP1 in our human tissue could be saturated and no further effect of exogenous SFRP1 could be observed or because we were comparing macrophages in a tissue versus bulk purified and stimulated macrophages.

In solid tumors, 5–40% of the tumor mass consists of tumor-associated macrophages (TAMs) and poor prognosis is associated with elevated levels of TAMs [44]. Stimuli in the tumor environment polarize TAMs towards a protumor M2 rather than an anti-tumor M1 phenotype [45]. TGF-β promotes tumor progression by recruiting TAMs to compete with dendritic cells by suppressing their antigen-presentation [34]. Zhang et al. provide evidence that TGF-β blocks M1 macrophage development while promoting the activation of M2 macrophages [46]. The expression of Egr2 has recently been utilized to describe murine the M2 macrophages [19]. Our results add to these finding by showing that the addition of extracellular recombinant SFRP1 represses TGF-β stimulated M2 polarization. Moreover, human explant mammary gland cultures treated with rSFRP1 show a marked reduction in the human M2 marker, CD163. CD163 is a monocyte/macrophage-restricted scavenge receptor [47] and the mechanism by which rSFRP1 reduces CD163 expression may be due in part to repression of Wnt signaling because Bergenfelz et al. show that breast cancer CD163^+^ TAMs correlate with Wnt5a expression, which is responsible for macrophage reprogramming to an anti-inflammatory M2 status [48].

## Conclusions

Taken together, these observations may provide insight into the role SFRP1 plays in tumor susceptibility. SFRP1 levels are reduced with increasing age and diminished SFRP1 has been noted in atypical breast lesions [49, 50]. Our studies suggest that loss of SFRP1 in epithelial cells can enhance TGF-β mediated EGR2 expression and affect TGF-β induced M2 polarization of macrophages. As M2 macrophages secrete growth factors, such as Wnt ligands and EGF, this could contribute to tumor progression through a feed forward cross talk between the epithelium and the immune system.

## Additional files

Additional file 1: Figure S1. The effect of SFRP1 on EGR2 expression in Breast Cancer cells. (A) T47D, MDA-MB, and TMX2–28 cells were transfected with an SFRP1 expression plasmid as described in materials and methods. Total RNA was harvested and subjected to real-time PCR analysis of *EGR2* expression. The results shown represent experiments performed in duplicate and normalized to the amplification of *ACTB* mRNA. Bars represent mean ± SEM of the fold change with respect to vector transfected control cells. *p < 0.05, (significantly different from control using student’s *t*-test). (PDF 382 kb)

Additional file 2: Figure S2. The expression of *EGR*2 is up-regulated in response to TGF-β treatment in human and murine mammary epithelial cells. (A) TERT-pSUPER cells and (B) control MMECs were treated in triplicate wells in the absence and presence of 2.5 ng/mL TGF-β for 24 h. Total RNA was harvested and subjected to real-time PCR analysis of *EGR2* expression. The results shown represent experiments performed in duplicate and normalized to the amplification of *ACTB* mRNA. Bars represent mean ± SEM of the fold change with respect to untreated. *p < 0.05 (significantly different from control treated cells using student’s *t*-test). (PDF 491 kb)

Additional file 3: Figure S3 The effect of ERK1/2 inhibition on EGR2 expression in human and mouse mammary epithelial cells deficient in SFRP1 expression. (A) TERT-pSUPER and TERT-siSFRP1 cells were treated with 10 μM FR108204 for 24 h and total RNA was isolated from three separate harvests for real-time PCR analysis of *EGR*2. (B) Mouse mammary epithelial cells were treated with 10 μM FR108204 for 24 h and total RNA was isolated from three separate harvests for real-time PCR analysis of *Egr*2. All real-time PCR results are from two separate experiments performed in triplicate and results were normalized to amplification of *ACTB* mRNA. Bars represent mean ± SEM and are expressed as fold change with respect DMSO treated cells. *p < 0.05, **p < 0.01 (significantly different from DMSO treated group using student’s *t*-test). (PDF 163 kb)

## Abbreviations

CD163: CD 163 molecule
EGR2: Early Growth Response 2
ERK1/2: Extracellular-signal-regulated kinase1/2
Gpr18: G protein-coupled receptor 18
HLA-DRA: Major histocompatibility complex, Class II, DR alpha
HMG: high mobility group
LDL: Low-density lipoprotein
SFRP1: Secreted frizzled-related protein-1
TAM: Tumor Associated Macrophage
TGF-β: Transforming growth factor-β
