# Supplementary figures and images for: Regulation of early growth response 2 expression by secreted frizzled related protein 1
Source: BMC Cancer. 2017 Jul 7;17:473. doi: 10.1186/s12885-017-3426-y (PMC5501954; doi:10.1186/s12885-017-3426-y)

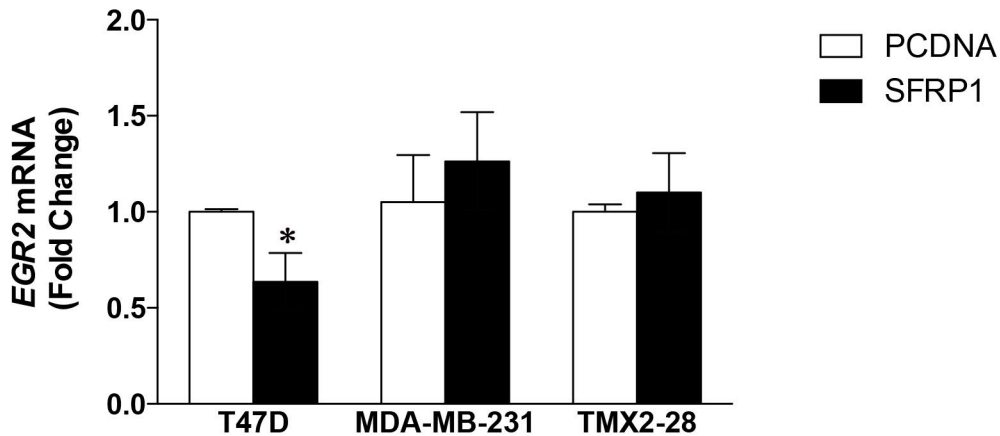

Supplemental Figure 1

Supplement: Supplementary file 1 — The effect of SFRP1 on EGR2 expression in Breast Cancer cells. (A) T47D, MDA-MB, and TMX2–28 cells were transfected with an SFRP1 expression plasmid as described in materials and methods. Total RNA was harvested and subjected to real-time PCR analysis of EGR2 expression. The results shown represent experiments performed in duplicate and normalized to the amplification of ACTB mRNA. Bars represent mean ± SEM of the fold change with respect to vector transfected control cells. *p < 0.05, (significantly different from control using student’s t-test). (PDF 382 kb) [file 12885_2017_3426_MOESM1_ESM.pdf]

**A**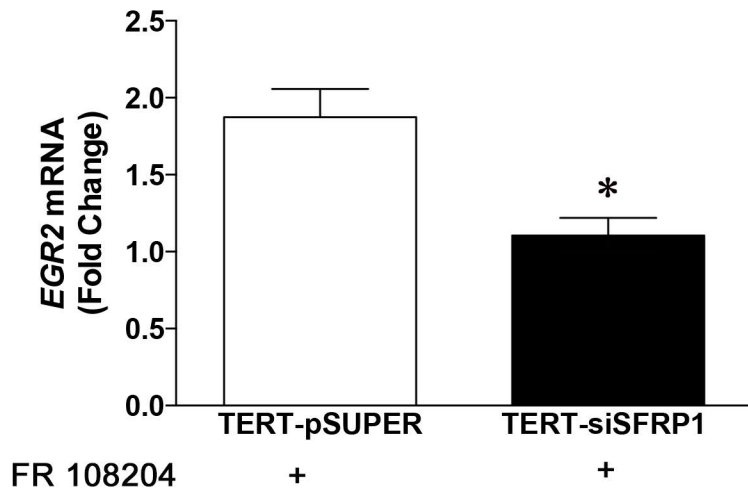**B**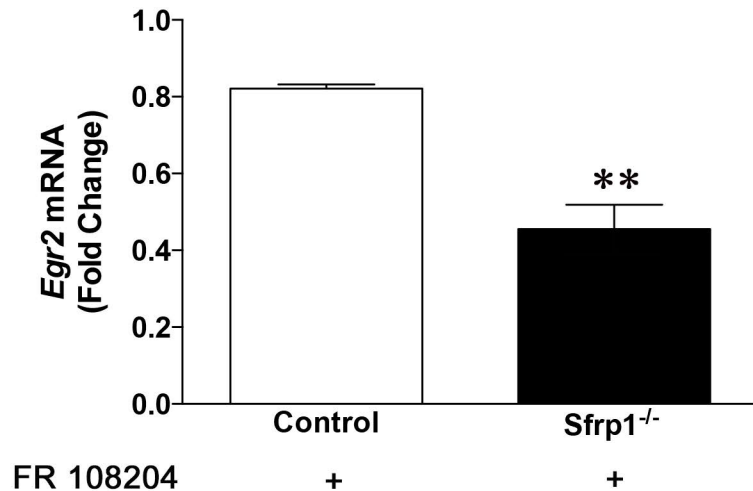

Supplemental Figure 3

Supplement: Supplementary file 2 — The expression of EGR2 is up-regulated in response to TGF-β treatment in human and murine mammary epithelial cells. (A) TERT-pSUPER cells and (B) control MMECs were treated in triplicate wells in the absence and presence of 2.5 ng/mL TGF-β for 24 h. Total RNA was harvested and subjected to real-time PCR analysis of EGR2 expression. The results shown represent experiments performed in duplicate and normalized to the amplification of ACTB mRNA. Bars represent mean ± SEM of the fold change with respect to untreated. *p < 0.05 (significantly different from control treated cells using student’s t-test). (PDF 491 kb) [file 12885_2017_3426_MOESM3_ESM.pdf]

**A**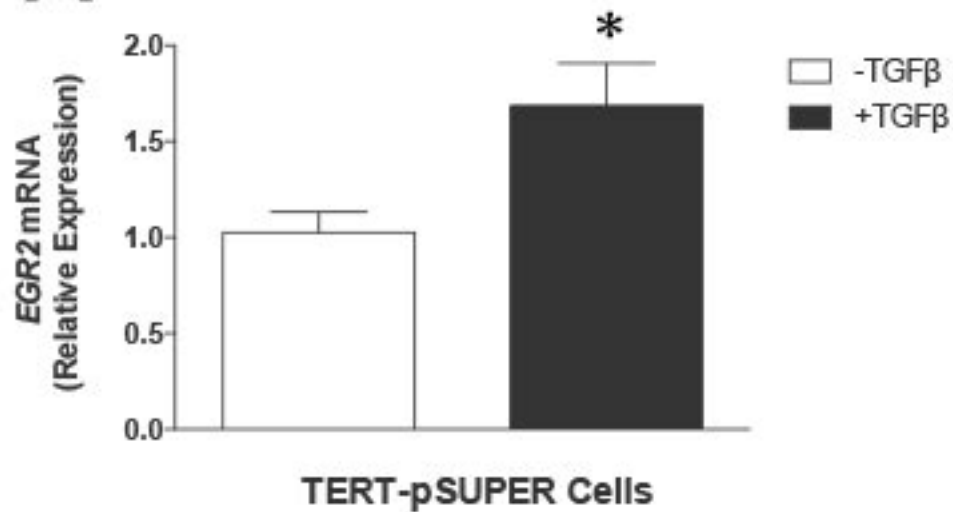**B**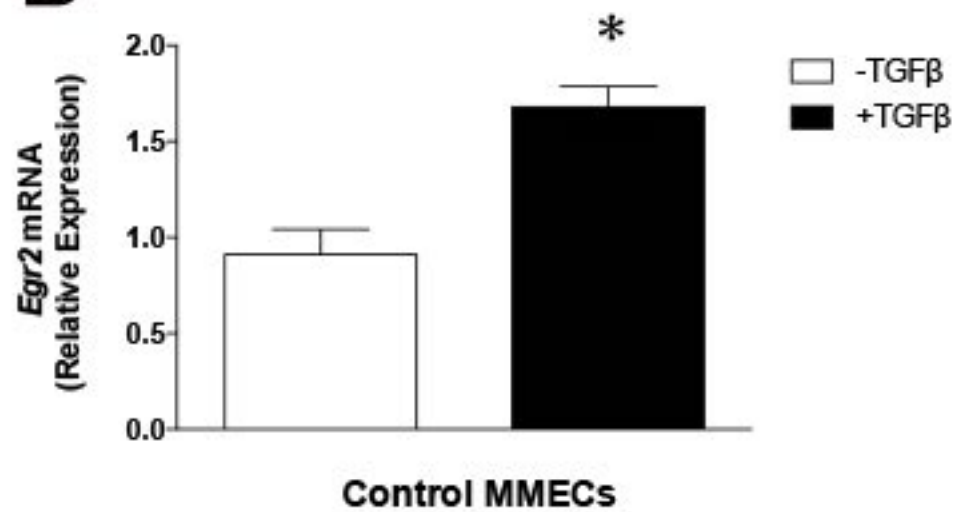

Supplemental Figure 2

Supplement: Supplementary file 3 — The effect of ERK1/2 inhibition on EGR2 expression in human and mouse mammary epithelial cells deficient in SFRP1 expression. (A) TERT-pSUPER and TERT-siSFRP1 cells were treated with 10 μM FR108204 for 24 h and total RNA was isolated from three separate harvests for real-time PCR analysis of EGR2. (B) Mouse mammary epithelial cells were treated with 10 μM FR108204 for 24 h and total RNA was isolated from three separate harvests for real-time PCR analysis of Egr2. All real-time PCR results are from two separate experiments performed in triplicate and results were normalized to amplification of ACTB mRNA. Bars represent mean ± SEM and are expressed as fold change with respect DMSO treated cells. *p < 0.05, **p < 0.01 (significantly different from DMSO treated group using student’s t-test). (PDF 163 kb) [file 12885_2017_3426_MOESM2_ESM.pdf]
